# Clinical and epidemiological profiles of pediatric COVID-19 in two private Kenyan hospitals

**DOI:** 10.11604/pamj.2024.48.48.39305

**Published:** 2024-06-07

**Authors:** Del-rossi Sean Quadros, Jasmit Shah, Angela Migowa, Pauline Samia, William Macharia

**Affiliations:** 1 Department of Pediatrics and Child Health, Aga Khan University Hospital, Nairobi (AKUHN), Nairobi, Kenya,; 2 Department of Internal Medicine, Aga Khan University Hospital, Nairobi (AKUHN), Nairobi, Kenya,; 3 Department of Public Health and Primary Care, Ghent University, Ghent, Belgium

**Keywords:** Pediatric COVID-19, SARS-CoV-2, African children, clinical laboratory, radiological presentation

## Abstract

**Introduction:**

COVID-19 infection has attracted global attention with limited published data on the burden in African children.

**Methods:**

hospital-based longitudinal survey in children with COVID-19 infection, aged 0-18 years admitted between August 2020 and December 2021. The main objective of the study was to describe socio-demographic, clinical and diagnostic manifestations of COVID-19 infection in children.

**Results:**

the study enrolled 85 children. Median age was 5•1 years (IQR = 1•3 - 12•4) with equal gender distribution. Under five years were 52•9%. Average length of hospital stay among non-severe cases was three days (IQR=2•0-5•0). No deaths were reported. Fifteen patients (18•7%) were asymptomatic. The most common presenting symptoms were fever (51•8%), vomiting (36•5%), cough (27•1%), diarrhea (20•0%), nasal congestion (14•1%) and fast breathing (12•9%). Two patients presented in shock and features consistent with Multisystemic Inflammatory Syndrome in Childhood (MIS-C). Procalcitonin and C-reactive proteins were elevated in 76•9% and 45•8% respectively. Majority (n=80) had white cell counts within normal range and none had bacterial pathogens isolated from blood (n=63). Liver and Renal function tests were within the normal range in the majority of those tested (n=24 and n=64 respectively). Three of the five patients with elevated platelet count (>500 x109/L) had clinical diagnosis of MIS-C. Eight of 20 patients subjected to imaging had radiological features of bilateral ground glass opacifications while six of nine patients who presented with cardiovascular compromise had mild to moderate ventricular dysfunction on echocardiography.

**Conclusion:**

our study suggests that children in the African setting manifest a mild form of the COVID-19 infection with low mortality.

## Introduction

The novel coronavirus disease 2019 (COVID-19), is caused by the Severe Acute Respiratory Syndrome coronavirus-2 (SARS-CoV-2), a member of the beta coronaviruses [1]. COVID-19 was first detected in early December 2019 where pneumonia-like cases of unknown origin were initially reported in China with rapid global transmission. The emergence of SARS-CoV-2 has attracted global attention, with the World Health Organization (WHO) declaring COVID-19 a pandemic and a global health emergency in March 2020 [2,3]. Most attention on COVID-19 research has to date largely focused on severe respiratory manifestations in adults, with limited data on presentation in children [4]. Initial reports on laboratory confirmed COVID-19 patients revealed absence of cases in children below the age of fifteen [5]. Soon after, reports emerged describing children and newborns with COVID-19, indicating that the disease does not spare any age group [6-12]. The first confirmed pediatric patient was reported from China on January 20^th^, 2020 in a 14-year-old who died from the disease on February 7^th^, 2020. The youngest reported was of a newborn baby who tested positive just minutes after birth in London. Seven (7) Kenya reported the first pediatric case of COVID-19 in a six-year-old on March 12^th^ 2020, at a national referral hospital in early April 2020. The child unfortunately died from COVID-19 related complications a few days after the diagnosis. As of 4^th^ February 2022, more than 320,000 cases of COVID-19 infection had been recorded in Kenya out of which 5% were between 0-19 years [13]. COVID-19 pandemic continues to evolve as its impact, particularly among children and young adults, remains largely understudied. This study will help in improving the understanding of presentation of COVID-19 in children in low resource settings to prioritize use of limited diagnostic supplies in confirming or excluding the diagnosis. This may also help in early isolation and management.

## Methods

The primary objective was to describe the social-demographic, clinical, laboratory and diagnostic imaging findings of PCR, antigen or antibody confirmed COVID-19 infection in children in an African setting.

**Study design and setting:** this was a hospital based longitudinal survey of cohort of pediatric patients with COVID-19 infection, aged 0-18 years, admitted to two private healthcare referral facilities in Nairobi County between August 2020 and December 2021. The first, is a 254-bed private hospital providing general and specialised healthcare and the second, a 100-bed private pediatric hospital.

**Eligibility criteria:** all in-patients, aged 0-18 years, admitted with laboratory confirmed SARS-CoV-2 were enrolled into the study. The clinically suspected cases with a negative PCR, Antigen test and antibody test for SARS-CoV-2 were excluded.

**Data collection and analysis:** all patients admitted were screened for COVID-19 using both SARS-CoV-2 PCR (RealStar® SARS-CoV-2 RT-PCR Kit 1.0, Altona Diagnostics, diagnostic sensitivity of 100% and specificity of 96%) and SARS-CoV-2 antigen tests (PanbioTM COVID-19 Ag Rapid Test Device, Abbott Rapid Diagnostics, diagnostic sensitivity of 98•1% and specificity of 99•8%). All the cases were evaluated and managed by the primary treating paediatrician in consultation with the hospital's infectious disease team according to the hospitals policies for COVID-19 management. All laboratory and radiological tests were requested at the discretion of the primary admitting physician.

Medical records of the patients, at the study health facilities, who fulfilled the inclusion criteria, underwent detailed chart review. The data were captured electronically and stored in the Research Electronic Data Capture (REDCap) platform located at the Aga Khan University Hospital Nairobi. All patients were followed up from admission to discharge or transfer to other facilities. Each patient had a unique identifier from the parent institution and another for REDCap. Categorical data was presented as frequencies and percentages and continuous data was presented as medians and interquartile ranges. All the data was analysed using the IBM Statistical Package for the Social Sciences (SPSS) version 22•00.

**Ethical considerations:** approvals for the study were obtained from the Institutional Ethics Review Committee at the Aga Khan University Hospital Nairobi (Ref: 2020/IERC-91/v2, 19/08/2020), and Gertrude Children´s Hospital (Ref: GCH052/2020, 12/08/2020). Research permit was obtained from the National Commission for Science, Technology and Innovation (NACOSTI) (Ref: 432812, 28/08/2020) prior to study commencement. Stored data access was limited to the principal investigator and co-investigators.

## Results

This study was conducted between August 2020 and December 2021. From 4245 pediatric admissions, 85 patients tested positive for COVID-19 (2•0%) and were recruited and enrolled into the study. All admitted patients were followed up from admission to discharge or transfer from hospital. The median age of the participants was 5•1 years (IQR = 1•3-12•4). More than half (52•9%) were < 5 years with an equal gender distribution (50•6% boys, 49•4% girls). Majority (92•9%) of the patients were from families within the middle socio-economic status (gross monthly income estimated at 100,000-500,000 Kenya shillings) and whose parents had a formal education level beyond primary level (75•3%) (Table 1).

**Table 1:** general characteristics and demographics of paediatric patients admitted with COVID-19

Variable n = 85	No. (%)
**Sex**	
Male	43 (50.6)
Female	42 (49.4)
Age (years) (median [IQR])	5.1 (1.3-12.4)
<= 5 years	45 (52.9)
6-12 years	19 (22.4)
>= 13 years	21 (24.7)
**Family socio-economic status**	
Low (GMI <1,000 USD)	6 (7.1)
Middle (GMI 1,000-5,000 USD)	79 (92.9)
High ( GMI >5,000 USD)	0 (0.0)
**Years of formal education of parent(s)**	
None	11 (12.9)
Primary School	10 (11.7)
High School	8 (9.4)
College	56 (65.9)
Ethnicity	
African	78 (91.7)
Others	7 (8.3)
**Admission to hospital in previous 3 months**	
No	82 (96.5)
Yes	3 (3.5)

GMI-gross monthly income; USD-United States Dollar

Table 2 summarizes the clinical symptoms as defined by the primary treating physician, present at the time of admission. The 17•6% asymptomatic patients were admitted for an elective surgical or diagnostic procedure. The most commonly presenting symptoms were fever (51•8%, 95% CI 40•6%-62•7%), vomiting (36•5%, 95% CI 26•3%-47•6%), cough (27•1%, 95% CI 18•0%-37•8%), diarrhea (20%, 95% CI 11•3%-29•1%), nasal congestion (14.1%, 95% CI 7•5%-23.4%) and fast breathing (12.9%, 95% CI 6•6%-22•0%). From the 85 patients who tested positive for COVID-19, 26 (30.6%) patients had a history of previous contact exposure to a person known to have COVID-19. History of underlying chronic conditions was elicited in 23.5%. A pre-existing neurological disorder was reported by four patients (4.7%), three (3.5%) had a congenital heart condition, three (3.5%) with underlying liver pathology and another three (3.5%) with a chronic renal disorder while two (2.4%) patients had a malignant disorder and another 2 (2.4%) with a hematological disorder. Only one (1.2%) patient had a preexisting rheumatological disorder (Table 3).

**Table 2:** clinical symptoms of COVID-19 paediatric patients at admission

Variable n = 85	No. (%)
Asymptomatic	15 (17.6)
Fever (>38°C)	44 (51.8)
**Duration of fever (n=44)**	
< 3 days	29 (65.9)
4-6 days	8 (18.2)
>7 days	7 (15.9)
Abdominal Pain	15 (17.6)
Diarrhoea	17 (20.0)
Vomiting	31 (36.5)
Chest Pain	6 (7.1)
Fast breathing	11 (12.9)
Sore throat	8 (9.4)
Runny Nose	12 (14.1)
Seizures	7 (8.2)
Headache	12 (14.1)
Abdominal distension	1 (1.2)
Bloody stools	1 (1.2)
Constipation	1 (1.2)
Cyanosis	1 (1.2)
Jaundice	1 (1.2)
Joint pain	2 (2.4)
Palpitations	3 (3.5)
Fatigue	1 (1.2)
Photophobia	5 (6.3)
Poor feeding	1 (1.3)
Stridor	3 (3.8)
Syncope	1 (1.2)
Edema	1 (1.2)
Wheezing	2 (2.4)
Low urine output Rash	1 (1.2)

**Table 3:** underlying chronic conditions among COVID-19 paediatric patients at admission

Variable n = 85	No. (%)
Inflammatory/ Rheumatological disorders	1 (1.2)
Chronic Cardiac Disease	3 (3.5)
Asthma Other Chronic	2 (2.4)
Pulmonary Disease Malignant	0 (0.0)
Disorders Haematological	2 (2.4)
Disorders History of contact with confirmed COVID-19 Infection	2 (2.4)
Chronic Kidney	26 (30.6)
Disorders Chronic	3 (3.5)
Liver Disorders	3 (3.5)
Chronic Neurological	4 (4.7)
Disorders Congenital	0 (0.0)
Immune suppression	0 (0.0)
Diabetes/ Endocrine	0 (0.0)
Disorders	
Hypertension	

Table 4 summarizes the main physical findings seen in children admitted with COVID-19 infection. Most (90.6%) had a normal nutritional status with 6 (7.1%) being underweight. Dehydration (dry mucosal membranes, sunken eyes, depressed fontanelle) was noted in 25 children (29.4%). Two of the patients who presented in shock (delayed capillary refill time, low volume pulses, cool extremities) had other features consistent with MIS-C (Multisystemic Inflammatory Syndrome in Childhood) including a macular-papular rash and oral mucosal inflammation. Twenty-two patients (25.9%) had increased work of breathing, however with normal oxygen saturations of >97%. Eight patients (9.4%) had abdominal tenderness, with associated hepatomegaly seen in three of those patients. A small percentage of patients had neurological impairment with four (4.7%) being hypotonic and with abnormal reflexes and tone with associated delay in developmental milestones. Twenty-five (29.2%) presented with a high temperature (>37.2°C) and only six patients (7.1%) had a low oxygen saturation of < 92%.

**Table 4:** physical findings in COVID-19 paediatric patients at admission

	No. (%)
**Nutritional Status**	
Normal	77 (90.6)
Underweight	6 (7.1)
Overweight	2 (2.4)
**Oxygen Saturation (%)**	
>92	79 (92.9)
≤92	6 (7.1)
**Temperature (oC)**	
36.1-37.2 (normal)	60 (70.6)
≥37.3 (high)	25 (29.4)
Abnormal pulses	2 (2.4)
Abdominal tenderness	8 (9.4)
Hepatomegaly	3 (3.5)
Jaundice	2 (2.4)
Abnormal chest	10 (11.8)
sounds on auscultation	4 (4.7)
Neurological impairment	4 (4.7)
Rash	3 (3.5)
Oral muco-cutaneous inflammation	25 (29.4)
Dehydration Shock	2 (2.4)

Table 5 and Table 6 show the laboratory and imaging findings in children admitted with COVID-19 infection. A raised procalcitonin level was detected in 10 (n=13,76•9%) patients, while 27 (n=59,45•8%) had a raised CRP value and only one patient tested had a raised ESR level. Most of the patients (n=80, 62•5%) had a total white cell count within normal range, with no neutrophil or lymphocyte predominance. Most children had normal hepatic and renal function tests, all had normal coagulation profiles. A child with MIS-C had both a raised troponin and pro-BNP level. Three of the five patients with a raised platelet count were also diagnosed with MIS-C.

**Table 5:** laboratory findings in COVID-19 pediatric patients

Laboratory Markers	Median value (IQR)	Low No. (%)	Normal No. (%)	High No. (%)
Blood cell counts **Total white cell counts (x10/L) (n=80)**	6.9 ( 5.4-10.4)	27 (33.8)	50 (62.5)	3 (3.8)
**Neutrophils (x109/L) (n=80)**	3.5 (1.8-6.3)	72 (97.3)	2 (2.4)	-
**Lymphocytes (x109/L) (n=80)**	2.8 (1.9-3.9)	70 (100.0)	-	-
**Platelets (x109/L) (n=80)**	322.5 (250.0-388.0)	16 (20.5)	57 (73.1)	5 (6.4)
Biochemistry				
**Procalcitonin (ng/mL) (n=13)**	0.5 (0.1-2.7)	-	3 (23.1)	10 (76.9)
**CRP (mg/L) (n=59)**	4.0 (4.0-27.0)	-	32 (54.2)	27 (45.8)
**Haematocrit (%) (n=80)**	37.0 (35.0-41.0)	1 (1.8)	46 (80.7)	10 (17.5)
**Hemoglobin (g/L) (n=80)**	12.7 (11.6-14.0)	2 (2.5)	52 (65.0)	26 (32.5)
**Creatinine (mmol/L) (n=64)**	37.0 (25.5-48.0)	17 (26.6)	42 (26.6)	5 (7.8)
**Sodium (mEq/L) (n=64)**	138.0 (135.0-139.0)	4 (6.3)	58 (92.1)	1 (1.6)
**Potassium (mEq/L) (n=64)**	4.4 (4.0-4.7)	2 (3.2)	46 (74.2)	14 (22.6)
**Urea (mmol/L) (n=64)**	3.4 (2.9-4.5)	1 (1.5)	59 (90.8)	5 (7.7)
**Chloride (mEq/L) (n=64)**	104.0 (101.0-108.0)	7 (12.1)	51 (87.9)	-
**Bicarbonate (mEq/L) (n=64)**	20.6 (18.0-23.3)	25 (58.1)	18 (41.9)	-
**Calcium (mmol/L) (n=12)**	2.3 (2.1-2.4)	6 (50.0)	6 (50.0)	-
**Magnesium (mmol/L) (n=9)**	0.8 (0.8-0.8)	1 (11.8)	7 (77.8)	1 (11.8)
**ALT (U/L) (n=24)**	32.0 (18.0-59.0)	-	15 (62.5)	9 (37.5)
**AST (U/L) (n=24)**	38.0 (24.5-48.0)	-	17 (70.8)	7 (29.2)
**Total Bilirubin (µmol/L ) (n=24)**	11.9 (7.0-19.0)	-	17 (81.0)	4 (19.0)
**Glucose (mmol/L) (n=14)**	4.5 (3.8-5.1)	2 (14.3)	10 (11.8)	2 (14.3)
**Troponins (ng/mL) (n=1)**	96.0	-	-	1 (100.0)
**Vitamin D (n=5)**	31.8 (22.0-48.0)	2 (40.0)	3 (60.0)	-
**Lactate (mmol/L) (n=2)**	5.4 (3.8-7.0)	-	-	2 (100.0)
**Albumin (g/dL) (n=15)**	42.0 (33.0-46.0)	6 (40.0)	9 (60.0)	-
**INR (n=3)**	1.1 (1.1-1.2)	-	3 (100.0)	-

CRP=C-reactive protein; ESR=erythrocyte sedimentation reaction; ALT=alanine transaminase; AST=aspartate transaminase; pro-BNP= B-type natriuretic peptide; INR=international normalized ratio; CT=computer tomography; MRI=magnetic resonance imaging

**Table 6:** imaging findings in COVID-19 pediatric patients

Clinical outcomes	N=85 No. (%)
Average length of hospital stay (days)	3•0 (IQR 2•0-5•0)
≤2 days	39 (44•7%)
3-7 days	36 (42•4%)
≥8 days	11 (12•9%)
Admission to Intensive Care Unit	6 (7•1%)
Discharged Home	83 (97•6%)
Transfer to other facility	2 (2•4%)
Death	0 (0•0%)

A quarter of the patients, presenting with respiratory distress or oxygen saturation < 92% at admission had a chest radiography done. Eight patients had radiological abnormalities of bilateral ground glass opacifications. Two patients, requiring invasive mechanical ventilation, had a chest CT scan and both were reported to have bilateral ground-glass appearance. Nine patients presenting with cardiovascular compromise at admission had an echocardiography study done. Six of them had mild to moderate ventricular dysfunction while three had features of pericarditis and none manifested with coronary abnormalities. The three cases with pericarditis demonstrated cardiac valvular insufficiency of the tricuspid and mitral valves, unknown from previous history, and these cases were also associated with clinical features consistent with MIS-C. All the blood cultures done (n=63, 74•0%) for bacterial pathogens were negative at 72 hours of reporting.

Ninety-seven percent of the patients (n=85) had a positive PCR test, while 18% (n=44) had a SARS-CoV-2 positive antigen test. Using the PCR test as gold standard, the sensitivity and specificity of the COVID-19 antigen test in the study population were 87•5% (95% CI 47.3-99.7) and 5•6% (95% CI 0•7-18•6) respectively. Three patients with clinical diagnosis of MIS-C had a negative PCR test and a positive SARS-CoV-2 antibody test, demonstrating that MIS-C is a late complication of COVID-19 infection as opposed to an acute one. Table 7 shows the hospitalization status of the COVID-19 pediatric patients admitted. Among the 85 patients admitted, only six (7.1%) required admission to the critical care units, with three (3.5%) needing invasive mechanical ventilation as well as inotropic support. The average length of hospital stays among the stable patients admitted to the general isolation wards was three days (IQR 2-5) while two patients had an ICU stay of >7 days and one patient had an ICU stay of 44 days before transfer to another facility. Majority (83•0%) of the patients admitted for COVID-19 infection were discharged from hospital with no clinical sequelae. The three patients admitted to ICU requiring mechanical ventilation and inotropic support had presenting clinical features consistent with MIS-C. Of note, there were no deaths reported among this cohort of patients managed for COVID-19.

**Table 7:** hospitalization status of COVID-19 paediatric patients

Clinical outcomes	N=85 No. (%)
Average length of hospital stay (days)	3•0 (IQR 2•0-5•0)
≤2 days	39 (44•7%)
3-7 days	36 (42•4%)
≥8 days	11 (12•9%)
Admission to Intensive Care Unit	6 (7•1%)
Discharged Home	83 (97•6%)
Transfer to other facility	2 (2•4%)
Death	0 (0•0%)

## Discussion

In this study, we found an overall prevalence of pediatric COVID-19 below 1%, median age of 5.1 years (IQR = 1.3-12.4), 52.9% below the age of 5 years, 23.4% in the 6-12-year bracket and 24•7% being over 13 years of age. The initial review of positive COVID-19 cases, by the *Chinese Novel Coronavirus Pneumonia Emergency Response Epidemiological Team*, was a report of 72,314 subjects, that found about 2.0% of all the 44,672 COVID-19 confirmed cases were children aged 0-19 years, of which 0.9% were below 10 years [8]. Dong *et al*. in a review of 2,143 cases, looking at the epidemiology of COVID-19 among children reported a median age of seven years at presentation [6]. Similar findings were reported in a systematic review by Nachega *et al*. [14] and recently by Omar Irfan *et al*. [12] who reported a mean age of seven years. Data from these reports confirmed that children of all ages are at risk of contracting COVID-19, and there is no gender difference. The largest known review in Africa, by Nachega *et al*. report 469 children admitted with COVID-19 with a mean age of 5.9 years [14]. Local data, as of 4^th^ February 2022 from the Kenyan Ministry of Health (MOH), confirmed 321,922 cases of COVID-19 infection, of these approximately 5% are within the bracket of 0-19 years of age with no gender dominance [13].

We report a mild form of clinical presentation of COVID-19 infection, where 15 (18.7%) patients were asymptomatic. The most common presenting symptoms were fever (51.8%, 95% CI 40.6%-62.7%), vomiting (36•5%, 95% CI 26•3%-47•6%), cough (27.1%, 95% CI 18.0%-37.8%), diarrhea (20%, 95% CI 11.3%-29.1%), nasal congestion (14.1%, 95% CI 7.5%-23.4%) and fast breathing (12.9%, 95% CI 6.6%-22.0%). These presenting symptoms lasted 1-3 days with majority of the cases (92•9%) being clinically stable and eventually discharged with no clinical sequelae, only six cases (7•1%) required critical care support. A multi-country African report by Nachega *et al*. [14] reported similar clinical presentation with cough (37.0%), fever (31.0%), Rhinorrhea (25.1%) and respiratory distress (23.2%) as the most common symptoms. Unlike adults, children infected with COVID-19 tend to be asymptomatic [12,15-17]. Non-specific symptoms such as fever, lasting for 1-2 days, and cough are the most common clinical symptoms reported [15,18,19]. Other symptoms include mild upper respiratory tract symptoms, headache, myalgia, fatigue, abdominal pain, vomiting and diarrhea [15,20-21]. A few case reports demonstrate that gastrointestinal symptoms could be the initial symptoms [16,17] with other distinct symptoms such as irritability, poor feeding and decreased response being the only signs of COVID-19 infection in infants [17]. In the systematic review by Panahi *et al*. [15], clinical types of COVID-19 were grouped into three domains; mild, moderate and severe with the majority of cases being mild to moderate. Nachega *et al*. [14] reported that 52.5% presented with mild to moderate disease and 47.5% with severe disease requiring critical care support. Omar Irfan *et al*. [12] reported that 57.4% of cases required hospitalization, with majority (88.9%) recovering with no sequelae.

Recent reports have emerged of a previously undescribed presentation in children and adolescents reporting a multi-system inflammatory condition with a mix of signs and symptoms similar to those of Kawasaki Disease (KD) and Toxic Shock Syndrome (TSS), later termed as Multisystemic Inflammatory Syndrome in Childhood (MIS-C). In this study we report three cases (3•5%) of MIS-C that presented with shock and features of Kawasaki disease, all three cases required critical care support in form of mechanical ventilation and inotropic support. These cases all had a negative SARS-CoV-2 PCR test, with a positive SARS-CoV-2 antibody test, demonstrating past infection with COVID-19 rather than an acute infection, a similar finding reported previously [22,23]. This delayed presentation leads to multi-organ failure and shock. Although the true incidence is yet to be established, MIS-C appears to be a rare complication of the COVID-19 infection among children [14,22,23].

In our study, there was limited laboratory and imaging testing, as this was requested at the discretion of the primary treating physician, and attributed mainly to the mild clinical presentation. Elevated inflammatory markers (neutrophils, CRP, procalcitonin, ESR) were noted among the more severe cases admitted to the Intensive Care Unit (ICU). Markers of organ dysfunction (urea, creatinine, liver enzymes and cardiac enzymes) were elevated in the more critical patients, especially those diagnosed with MIS-C, with one of these patients having both a raised troponin and pro-BNP level. Three of the five patients with a raised platelet count had also been diagnosed with MIS-C. Majority of the imaging (chest X-ray and CT scan, echocardiography) was done for the more severe COVID-19 positive patients that demonstrated either respiratory or cardiovascular clinical decompensation.

Unlike adults with mild to severe COVID-19 infection, where laboratory abnormalities have been widely reported and somehow consistent, little is known about the laboratory profiles of children with COVID-19, with majority of the initial published data stemming from case reports and case series. Henry B *et al*. [24] in a pooled analysis of 24 studies, mostly from China, described the changes in 27 different laboratory parameters. The laboratory abnormalities in mild COVID-19 was seen as a decreased neutrophil count, with a pooled prevalence estimate (PPE) of 38•0% (95% CI 19.0-60.0%). Elevation in C-Reactive Protein (CRP), Procalcitonin (PCT) and Lactate Dehydrogenase (LDH) were also seen with PPEs of 18.0%, 26.0% and 28.0% respectively. Creatinine-Kinase-MB (CK-MB) was also elevated in one-third of patients (PPE, 33.0%; 95% CI 25.0-42.0%). Only two studies reported on cytokines with Interleukin-10 (IL-10) being the most frequently elevated cytokine (75.0%) and IL-6 and Interferon gamma (IFN-gamma) were also elevated though in only 37.0% and 25.0% of cases respectively. Elevated CK-MB, D-Dimer and prothrombin time (PT) could also be seen in severe cases.

Most patients investigated radiologically for chest pathology were patients in the mild clinical category which requires a balance between the risk of radiation and necessity for radiation exposure. In our study, only two patients, requiring invasive mechanical ventilation, had a chest CT scan and both showed bilateral ground-glass appearance. Chest Computer Tomography (CT) can detect lung abnormalities before RT-PCR assay turning up positive, even in asymptomatic carriers [18,25]. Duan YN *et al*. [26] in their study that assessed the chest CT changes among paediatric COVID-19 patients, concluded that, compared to adults, the chest CT characteristics of COVID-19 in children were atypical, with lower ground glass opacity attenuation with relatively rare interlobular septal thickening.

Although the pandemic focus is usually directed towards the economically productive age groups, the impact of COVID-19 on the paediatric population will be important to accurately model the pandemic and to ensure appropriate resources are allocated to those children requiring care. Many infectious diseases, including the SARS-CoV and MERS pandemics, affect children differently from adults, and a thorough understanding into those differences, especially now during the SARS-CoV-19 pandemic, will lead to important insights into the disease pathogenesis that will eventually guide in the development management policies and protocols. More documentation on clinical and epidemiological manifestations of pediatric-specific COVID-19 disease attributes among the African population, may enhance suspicion for early diagnosis, isolation and management in low income settings with limited laboratory resources.

## Conclusion

Our study suggests that children contract a mild form of the COVID-19 infection with a favorable outcome. The more severe cases, requiring critical care support, are part of the newly described complication post COVID-19 infection in children, known as the Multisystemic Inflammatory Syndrome in Childhood (MIS-C). Further investigation is needed both locally, regionally and globally on disease manifestation and the long-term complications related to COVID-19 in children.

### What is known about this topic

- *This study will help in improving the understanding of presentation of COVID-19 in children in low-resource settings to prioritize use of limited diagnostic supplies in confirming or excluding the diagnosis;*
- *This may also help in early isolation and management*.

### What this study adds

- *Although mild, further research on the role of children in transmission rate of COVID-19 is needed;*
- *Vaccination and community surveillance testing policy strategies may be targeted to more vulnerable groups*.
